# A novel Betaretrovirus discovered in cattle with neurological disease and encephalitis

**DOI:** 10.1186/s12977-021-00585-x

**Published:** 2021-12-20

**Authors:** Melanie M. Hierweger, Michel C. Koch, Ronja V. Kauer, Zoltán Bagó, Anna Oevermann, Giuseppe Bertoni, Torsten Seuberlich

**Affiliations:** 1 Division of Neurological Sciences, Department of Clinical Research and Veterinary Public Health, Vetsuisse Faculty, University of Bern, Bern, Switzerland; 2 Austrian Agency for Health and Food Safety (AGES) Ltd., Institute for Veterinary Disease Control, Mödling, Austria; 3 Institute of Virology and Immunology, Bern, Switzerland and Department of Infectious Diseases and Pathobiology, Vetsuisse Faculty, University of Bern, Bern, Switzerland

**Keywords:** Bovine retrovirus, Non-suppurative encephalitis, Neurological disease, Virus discovery, High-throughput sequencing, Cattle

## Abstract

**Background:**

The majority of emerging infectious diseases in humans are of animal origin, and many of them are caused by neuropathogenic viruses. Many cases of neurological disease and encephalitis in livestock remain etiologically unresolved, posing a constant threat to animal and human health. Thus, continuous extension of our knowledge of the repertoire of viruses prone to infect the central nervous system (CNS) is vital for pathogen monitoring and the early detection of emerging viruses. Using high-throughput sequencing (HTS) and bioinformatics, we discovered a new retrovirus, bovine retrovirus CH15 (BoRV CH15), in the CNS of a cow with non-suppurative encephalitis. Phylogenetic analysis revealed the affiliation of BoRV CH15 to the genus *Betaretrovirus.*

**Results:**

BoRV CH15 genomes were identified prospectively and retrospectively by PCR, RT-PCR, and HTS, with targeting of viral RNA and proviral DNA, in six additional diseased cows investigated over a period of > 20 years and of different geographical origins. The virus was not found in brain samples from healthy slaughtered control animals (n = 130). We determined the full-length proviral genomes from six of the seven investigated animals and, using in situ hybridization, identified viral RNA in the cytoplasm of cells morphologically compatible with neurons in diseased brains.

**Conclusions:**

Further screening of brain samples, virus isolation, and infection studies are needed to estimate the significance of these findings and the causative association of BoRV CH15 with neurological disease and encephalitis in cattle. However, with the full-length proviral sequences of BoRV CH15 genomes, we provide the basis for a molecular clone and further in vitro investigation.

**Graphical Abstract:**

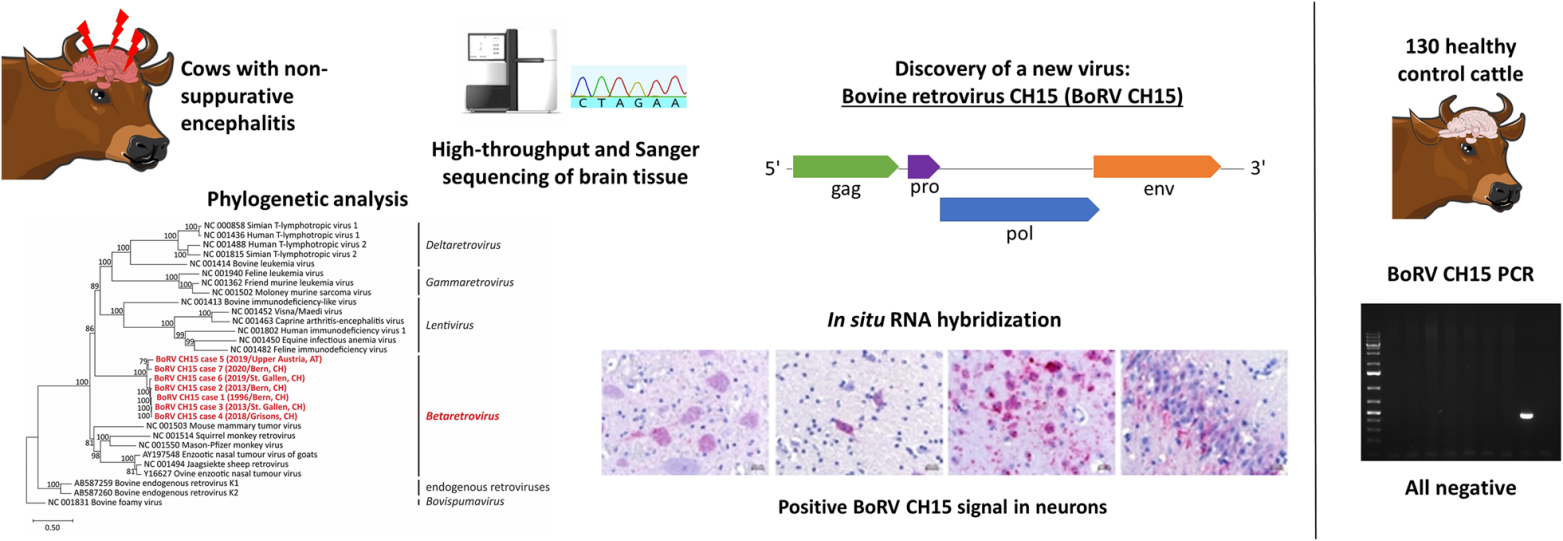

**Supplementary Information:**

The online version contains supplementary material available at 10.1186/s12977-021-00585-x.

## Background

The majority of emerging infectious diseases are of animal origin, and many emerging pathogens infect the central nervous system (CNS) [1–3]. Many encephalitis cases in animals remain etiologically unresolved, posing a constant threat to animal and human health [4, 5]. Due to the close contact between farm animals and humans, pathogens are easily transmitted between these hosts. Livestock also act as transitory hosts that are intermediate between wildlife and humans, for example in the case of Nipah virus in Malaysia [6, 7]. Thus, the extension of our knowledge of the repertoire of neuroinfectious viruses in livestock is important for the early identification of emerging diseases.

In retrospective screening studies of cattle with non-suppurative encephalitis performed with high-throughput sequencing (HTS) and bioinformatics, we have extended the known repertoire of neuropathogenic viruses [8–14]. In two of these studies, we discovered sequences of a putative new retrovirus, bovine retrovirus CH15 (BoRV CH15), in the CNSs of two cows with non-suppurative encephalitis and one cow with no lesion in the available brain tissue [8, 9]. A fourth animal was initially PCR positive, but was excluded from further analysis because the result was not confirmed upon retesting with different PCR assays. Phylogenetic analysis revealed an affiliation of BoRV CH15 to the genus *Betaretrovirus* [8]. However, at that point we could draw no conclusion regarding the association of BoRV CH15 infection with encephalitis in cattle.

Retroviruses are single-stranded positive-sense RNA viruses that incorporate their genomes as proviruses into host chromosomal genomes after reverse transcription to cDNA. Members of the family *Retroviridae* infect a huge range of host species [15]. In cattle, known retroviruses are bovine leukemia virus (BLV) of the genus *Deltaretrovirus*, bovine immunodeficiency virus (BIV) of the genus *Lentivirus*, and bovine foamy virus (BFV) of the genus *Bovispumavirus* [16]. BLV can lead to enzootic bovine leukosis and lymphoma [17, 18], whereas BIV and BFV are considered to be non-pathogenic in taurine cattle (*Bos taurus taurus*) [19, 20]. In bali cattle (*Bos javanicus domesticus*) however, jembrana disease virus, a close relative to BIV, can lead to severe disease as well [21]. All three viruses (BLV, BIV and BFV) can reach prevalences of up to 85% in certain geographic regions [22–24].

Several retroviruses, including members of the genera *Lentivirus*, *Gammaretrovirus*, and *Deltaretrovirus* have been shown to cause disease in the CNS. Whereas lentiviruses such as human immunodeficiency virus 1 (HIV-1) and small ruminant lentiviruses infect invading macrophages and glia cells, and deltaretroviruses such as human T-lymphotropic virus 1 infect mainly invading lymphocytes, gammaretroviruses such as murine leukemia virus are additionally neuronotropic [25]. Given the wide distribution of bovine retroviruses and the known neuroinvasive potential of retroviruses, further investigation of the association of BoRV CH15 with bovine non-suppurative encephalitis is important.

We prospectively examined cattle with neurological clinical signs that were submitted to the Division of Neurological Sciences, University of Bern, Switzerland, for neuropathological investigation. We conducted HTS of total RNA extracted from brain samples from animals with non-suppurative encephalitis, from which known neurotropic viruses (rabies virus, ovine herpesvirus 2, bovine astroviruses, and, in some cases, flaviviruses) were excluded. By these means, we identified BoRV CH15 in the brains of four additional animals. We determined the entire proviral genome of BoRV CH15, demonstrated its viral replication in situ, and assessed the causal associations between infection with this virus and the development of neuropathological lesions and disease.

## Results

### Case information

The BoRV CH15–positive cattle investigated in this study (cases 1–7) originated from different regions of Switzerland and Austria, and were identified by postmortem neuropathological diagnostics conducted over > 20 years (1996–2020) within the framework of surveillance programs for neuroinfectious diseases. Cases 1, 4, 5, 6, and 7 were clinically suspicious for bovine spongiform encephalopathy (BSE) and were thus submitted to reference laboratories (= BSE suspect cases). Cases 2 and 3 died on the farm or were euthanized for reasons other than human consumption (= fallen stock). All animals were dairy or suckler cows of various breeds. These animals were relatively old, with ages ranging from 8 to 18 years. In case 3, information about clinical signs was not available. In the remaining cases, neurological signs were reported, but with variable manifestations and severities. In animals with reported neurological signs, non-suppurative encephalitis was diagnosed by histopathological examination (Table 1).

**Table 1:** Overview of bovine retrovirus CH15–positive cases

Case # /Animal ID	Year	Location (Country)	Breed	Age (years)	Neurological signs	Neuropathology
**1/**25018	1996	Bern (CH)	n.k	8	Yes (not further defined)	Non-suppurative encephalitis
**2**/47417	2013	Bern (CH)	Simmental	11	Yes (recumbency)	Non-suppurative encephalitis
**3**/47621	2013	St. Gallen (CH)	Brown Swiss	18	n.k	No brain lesion
**4**/51185	2018	Grisons (CH)	Limousin	8	Yes (aggressiveness, nervousness, movement disorders)	Non-suppurative encephalitis
**5**/19140387	2019	Upper Austria (AT)	Fleckvieh	10	Yes (nervousness, overexcitability, reduced vision, seizure-like movement disorders)	Non-suppurative encephalitis
**6**/51410	2019	St. Gallen (CH)	Limousin	9	Yes (overexcitability, mandibular shaking, recumbency, loss of appetite)	Non-suppurative encephalitis
**7**/60162	2020	Bern (CH)	Tyrol Grey	9	Yes (head shaking, recumbency)	Non-suppurative encephalitis

CH, Switzerland; n.k., not known; AT, Austria

### Molecular diagnostics and sequencing results

The determination of the coding-complete BoRV CH15 genomes in cases 1 and 2 using HTS has been described previously [8, 9]. The resulting scaffolds showed a typical retrovirus genome structure with open reading frames (ORFs) for the putative group-specific antigen (gag), the putative protease (pro), the putative reverse transcriptase, RNase H and integrase (pol), and the putative envelope protein (env), and with sequence similarities of encoded proteins to members of the genus *Betaretrovirus*. For the remaining cases, diagnostic HTS was performed on brain-tissue RNA extracts. Generated read numbers ranged from ~ 73 Mio to ~ 150 Mio; in cases 4–7, nearly the full BoRV CH15 coding sequence was covered (Additional file 1). Remaining sequencing gaps were filled by RT-PCR and subsequent Sanger sequencing to obtain coding-complete BoRV CH15 genomes.

HTS of case 3 did not yield scaffolds with similarities to BoRV CH15 in our de novo assembly pipeline, which analyzes scaffolds of > 499 base pairs (bp). However, mapping of the reads to the BoRV CH15 genome showed that ~ 30% of the genome was covered, with reads mapping primarily to seven regions of 130–280 bp (three in the gag ORF, one in the pro ORF, two in the pol ORF, and one in the env ORF). Thus, viral RNA was present, but in a relatively small amount.

With rapid amplification of cDNA ends (RACE) on RNA in cases 1 and 2, we determined the authentic 3' end of the BoRV CH15 RNA genome in these cases. 5' RACE did not yield robust results.

To confirm that the BoRV CH15 genomes were integrated into the host genome, we performed PCRs targeting the gag, pol, and env ORFs in extracted DNA with previously published primer pairs [8]. In all cases, including case 3, all three PCR runs yielded clearly positive results, whereas those for non-template controls and negative control animals with non-suppurative encephalitis but BoRV CH15 negativity remained negative. We then determined the entire coding sequence of the BoRV CH15 proviral genome in brain tissue DNA extracts of case 3 by Sanger sequencing of overlapping PCR amplicons.

**Fig. 1:**
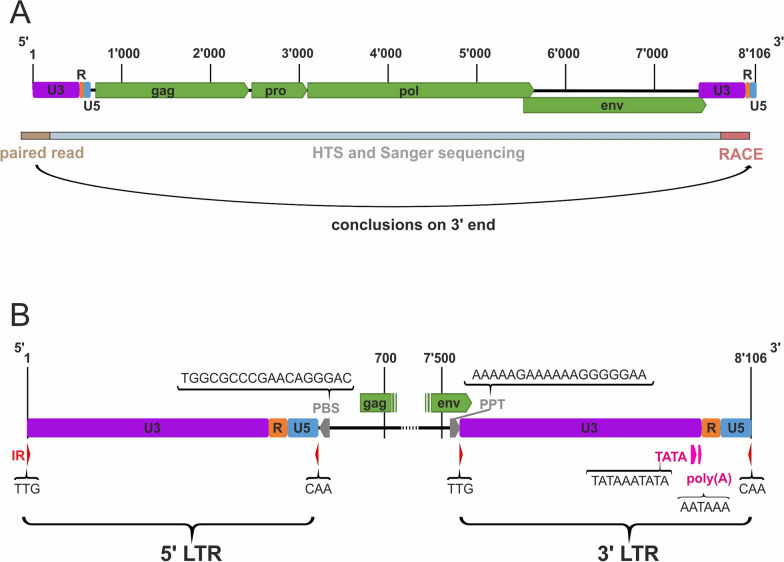
The bovine retrovirus CH15 proviral genome, sequencing strategy, and long terminal repeat (LTR) characteristics. **A** The coding-complete BoRV CH15 genome was determined by high-throughput sequencing (HTS) and Sanger sequencing. With 3' rapid amplification of cDNA ends (RACE), the LTR was sequenced to the redundant (R) region. With an HTS read pair overspanning the proviral 5' end and mapping to the *Bos taurus* genome, the missing bases of the 5' LTR could be determined. Because the LTRs have an identical sequence, the full-length proviral 3' LTR also could be determined. Green arrow-boxes represent the open reading frames of the group-specific antigen (gag), protease (pro), reverse transriptase, RNase H and integrase (pol), and envelope (env) proteins. Numbers represent bases in the viral genome. Purple, orange, and blue boxes depict the unique 3' (U3), R, and unique 5' (U5) regions of the LTRs, respectively. **B** Gray arrow-boxes represent the flanking regions of the LTRs: the primer-binding site (PBS) and polypurine tract (PPT). Pink boxes represent regulatory elements in the U3 region, the TATA-box (TATA) and the poly(A) signal (poly(A)). Inverted repeats (IR) are depicted by red triangles

To complete the long terminal repeats (LTRs) at the 5' and 3' ends of the proviral DNA genomes, sites of BoRV CH15 integration into the *Bos taurus* genome in case 1 were analyzed using the DNA HTS paired-end dataset generated previously. We identified six read pairs with one mate mapping to the *Bos taurus* genome (at six different sites) and the other mate mapping to the BoRV CH15 genome (Additional file 2). PCR amplification of regions between paired reads with read-specific primers failed. However, one HTS read bridged the 5' integration site (Additional file 2), which allowed us to determine the authentic 5' end of the integrated virus. With this information, we could establish the authentic 3' end of the integrated virus, as both LTRs of the provirus have identical sequences in retroviruses (Fig. 1A).

With primers binding the determined LTRs and the conserved primer-binding site (PBS) region, we were able to define the complete LTRs for the BoRV CH15 provirus genomes in cases 4–7. Some uncertainty remained regarding the LTRs of the BoRV CH15 genomes in cases 5 and 7. Sanger sequencing data suggest no uniform length for the integrated unique 3' (U3) region in these animals, with four possible insertions of 42–58 and 67–167 bp, respectively, in the BoRV CH15 genomes (Additional file 3). Insertions with good sequencing quality suggest duplications of U3 sequence elements. On blastn analysis, none of these inserts revealed a similarity to sequences of the bovine reference genomes or other available bovine sequences. As the LTRs did not show these insertions in the majority of PCR and Sanger sequencing runs of these cases, the proviral sequences without insertions in the LTRs were reported to GenBank.

Because the binding of cellular transcription factors is a basic principle in retrovirus replication [26, 27], the U3 regions of the LTRs in all cases were analyzed in silico for putative transcription factor–binding sites. This analysis revealed the presence of a nuclear factor 1 (NF-1) binding site shared by all of the Swiss BoRV CH15 sequences (Additional file 4). The sequence of the Austrian case 5 contained an insertion potentially disrupting this NF-1 site. An NF-1 site was also present in this sequence, but it was farther downstream than those in the Swiss sequences.

To assess sequence heterogeneity in different BoRV CH15 genomes (case 1 and cases 4–7), we remapped the raw reads to the complete genomes. Between 91 and 100% of the genomic sequences were covered by reads, with some uncovered nucleotides at the 5' and 3' ends in BoRV CH15 genomes in cases 1 and 4. The average coverage depth was > 65× and the average pairwise identities calculated over all reads per position was in all cases > 98.4%, indicating a high level of sequence similarity within the different animals.

### Bovine retrovirus CH15 strain genomes

In the seven BoRV CH15 strains sequenced, the proviral genomes ranged from 7′661 to 8′180 nucleotides (nt) in length, with a genomic organization typical of members of the genus *Betaretrovirus* (Table 2). All ORFs (gag, pol and env) were conserved and without apparent premature stop codons. Whereas the ORFs encoding the putative Gag, Pol, and Env proteins showed only minor variation in length, the pro-encoding ORF lengths ranged from 507 to 636 nt. The env and pol ORFs overlapped by 110 nt in all strains. We annotated the LTRs based on annotations published by Cousens et al. [28] for the enzootic nasal tumor virus. The total lengths were 348–607 nt. The U3 region lengths differed among viral genomes, ranging from 252 to 511 nt and containing important regulatory elements such as the TATA box and the poly(A) signaling sequence. The U3 region overlapped with the env ORF by 23 nt at the viral 3' end. In all BoRV CH15 genomes, the redundant (R) and unique 5' (U5) region lengths were 36 and 60 nt, respectively, and the LTR-flanking inverted repeats (IRs) were TTG and CAA. The LTRs were flanked by the PBS (TGGCGCCCGAACAGGGAC) at the 5' end and by the polypurine tract (PPT, AAAAAGAAAAAAGGGGGAA) at the 3' end (Fig. 1B).

**Table 2:** Putative genome-element lengths (nt) of seven bovine retrovirus CH15 strains

Region	Case 1	Case 2	Case 3	Case 4	Case 5	Case 6	Case 7
gag	1′731	1′731	1′731	1′731	1′731	1′731	1′728
pro	621	621	621	621	636	507	621
pol	2′562	2′562	2′562	2′562	2′562	2′565	2′562
env	2′064	2′064	2′064	2′064	2′067	2′064	2′067
U5	60	60	n.d	60	60	60	60
R	36	36	n.d	36	36	36	36
U3	474	511	n.d	273	359	452	252

gag, putative group-specific antigen open reading frame (ORF); pro, putative protease ORF; pol, putative reverse transcriptase, RNase H and integrase ORF; env, putative envelope ORF; U5, unique 5' region; R, redundant region; U3, unique 3' region; n.d., not determined

### Phylogenetic analysis and sequence comparison of bovine retrovirus CH15 strains

**Fig. 2:**
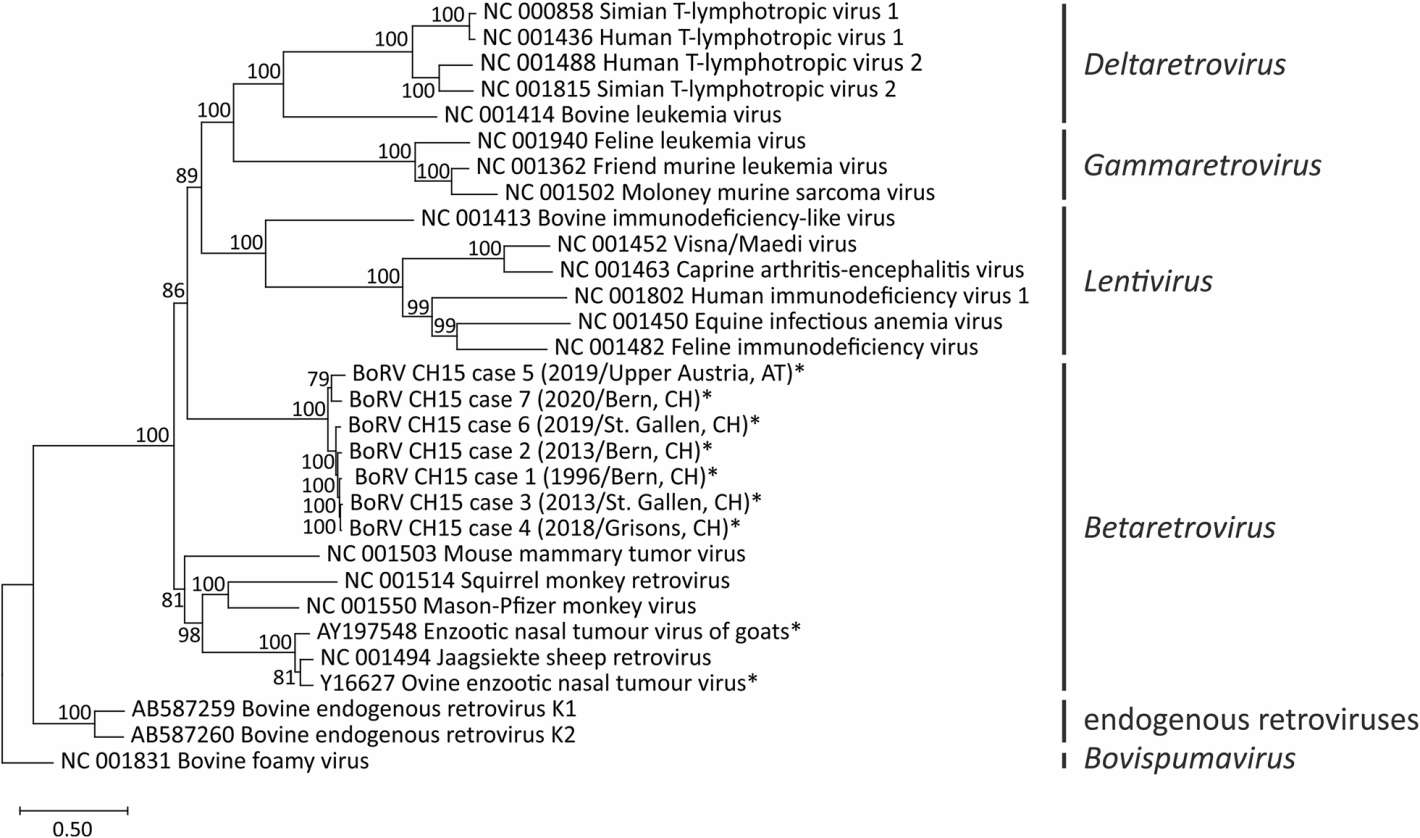
Phylogenetic analysis showed the affiliation of all bovine retrovirus CH15 genomes to the *Betaretrovirus* genus. The maximum-likelihood tree was based on selected coding-complete exogenous virus sequences of the genera *Betaretrovirus*, *Lentivirus*, *Gammaretrovirus*, *Deltaretrovirus,* and *Bovispumavirus,* as well as the env mRNA of bovine endogenous retroviruses. Branches are labeled by GenBank accession number and virus name. The scale indicates the number of substitutions per site, reflected by the branch lengths. Unclassified betaretroviruses are marked with an asterisk. CH, Switzerland; AT, Austria

Phylogenetic analysis was performed with the coding regions (start of the gag ORF to end of the env ORF) of selected exogenous virus genomes of the genera *Betaretrovirus*, *Lentivirus*, *Gammaretrovirus*, *Deltaretrovirus* and *Bovispumavirus*, as well as the env mRNA of two bovine endogenous retroviruses*.* In a maximum-likelihood phylogenetic tree, all BoRV CH15 genomes show an affiliation to the genus *Betaretrovirus *and neither to known neuroinvasive viruses belonging to genera *Lentivirus*, *Gammaretrovirus*, and *Deltaretrovirus*, nor to bovine endogenous retrovirus sequences (Fig. 2). Overall, the closest related virus was the Jaagsiekte sheep retrovirus (accession no. NC_001494), with sequence identities of 47.9–49.7% to the different BoRV CH15 strains. The BoRV CH15 genomes clustered closely together and showed an overall nucleotide identity of 88.5–98.8% to each other comparing the coding sequence (nucleotide sequence from the start of the gag ORF until the end of the env ORF) and an overall identity of the concatenated translated protein sequences of 91.9–99.2%. The genomes generated from cases 3 and 4 had the closest relationship and those generated from cases 5 and 6 were most diverse (Table 3). Comparison of individual ORF sequences and elements of the LTRs similarly reflected these relationships (Additional file 5).

**Table 3:** Pairwise sequence identity (%) of nucleotide coding sequences and concatenated protein sequences of bovine retrovirus CH15 strains [nt/aa]

Case	2	3	4	5	6	7
1	97.0/98.5	98.0/99.1	98.1/99.1	88.8/93.9	95.8/95.9	90.1/94.5
2		97.0/98.3	96.9/98.2	88.6/93.7	95.8/95.5	90.0/94.2
3			98.8/99.2	88.8/93.9	95.6/95.7	90.0/94.2
4				88.8/93.7	95.7/95.6	90.1/94.2
5					88.5/91.9	90.5/94.7
6						89.9/92.4

### Prevalence of bovine retrovirus CH15 in the brains of healthy slaughtered animals

To assess the association of BoRV CH15 infection to disease, 130 fresh-frozen medulla oblongata samples from healthy slaughtered cattle without histopathological lesions in the brain were tested for the presence of BoRV CH15 provirus. These control animals were of different breeds (Fleckvieh n = 51, Brown Swiss n = 32, Holstein n = 26, Simmental n = 6, Limousin n = 4, mixed breed n = 5, unknown breed n = 6) and had a mean age of 6 years. The minimum and maximum age of the control animals were 3 and 16 years, respectively, with the 1st quartile of 4 years and the 3rd quartile of 8 years. After DNA extraction, we assessed the DNA extraction efficiency and the presence of putative PCR inhibitors by qPCR targeting the bovine housekeeping gene 12s rDNA. DNA extracts had Cq values between 10.3 and 24.4 and thus extraction was evaluated as successful. On these DNA extracts (n = 130), we performed a PCR assay with a previously published primer pair amplifying a 500-bp-long fragment of the gag ORF [8] of BoRV CH15. All 130 DNA extracts were negative.

### Neuropathological features in bovine retrovirus CH15–positive animals

**Fig. 3:**
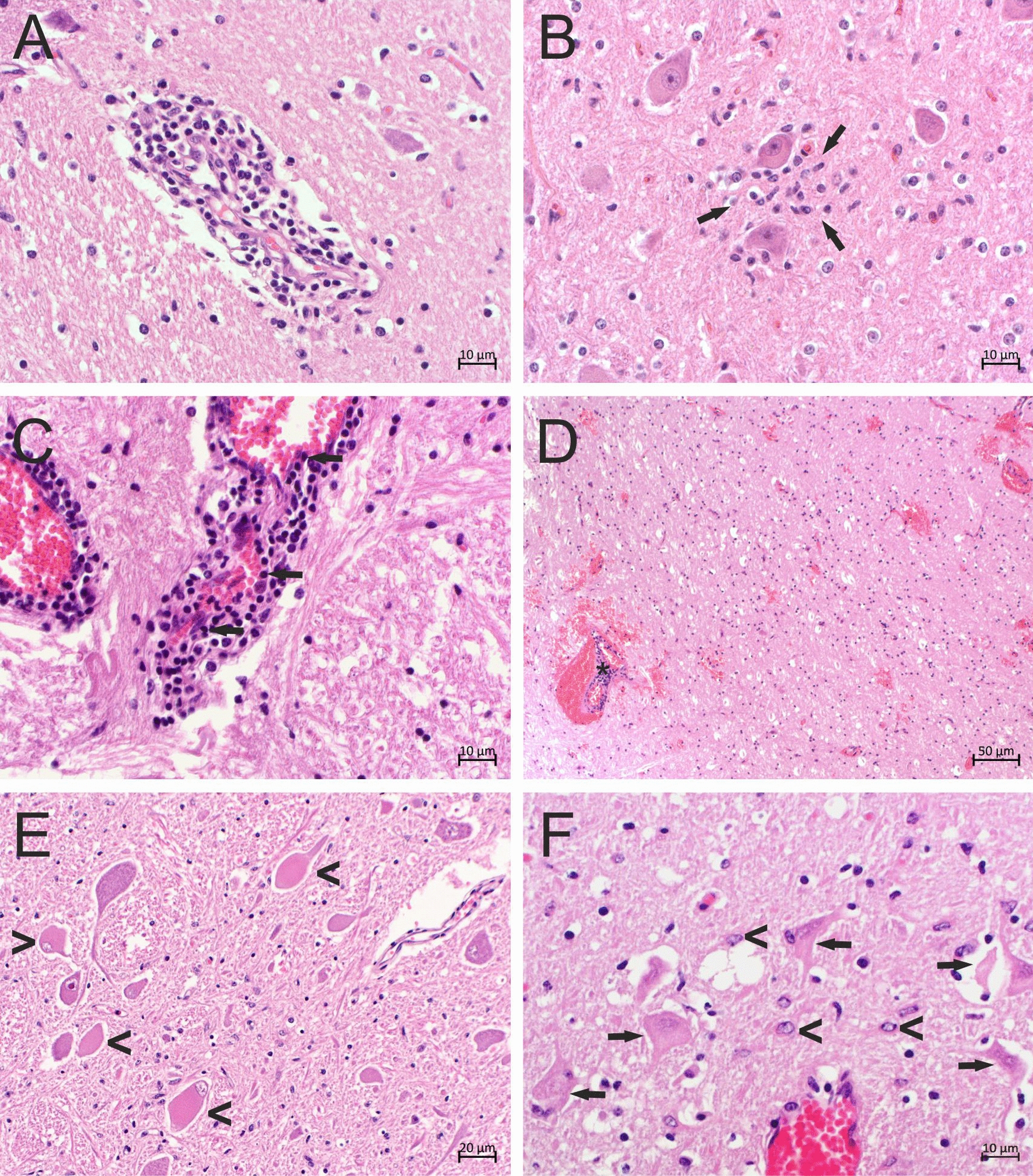
Bovine retrovirus CH15–positive animals showed non-suppurative encephalitis on histopathological examination. Formalin-fixed paraffin-embedded brain tissue slides were stained with hematoxylin and eosin. **A** Perivascular infiltrates of mononuclear cells forming a cuff around a vessel; case 6, hypothalamus. **B** Nodular gliosis (arrows) around neurons; case 1, medulla oblongata. **C** Perivascular cuff with infiltration of mononuclear cells in the vessel wall (arrows); case 7, medulla oblongata. **D** Severe ecchymotic and ring hemorrhages, often in association with perivascular cuffs (asterisk); case 7, thalamus. **E** Chromatolytic, swollen, and eosinophilic motoneurons (arrowheads); case 6, medulla oblongata. **F** Eosinophilic and shrunken pyramidal cells with loss of Nissl substance (arrows) surrounded by hypertrophic astrocytes (arrowheads); case 7, hippocampus

**Fig. 4:**
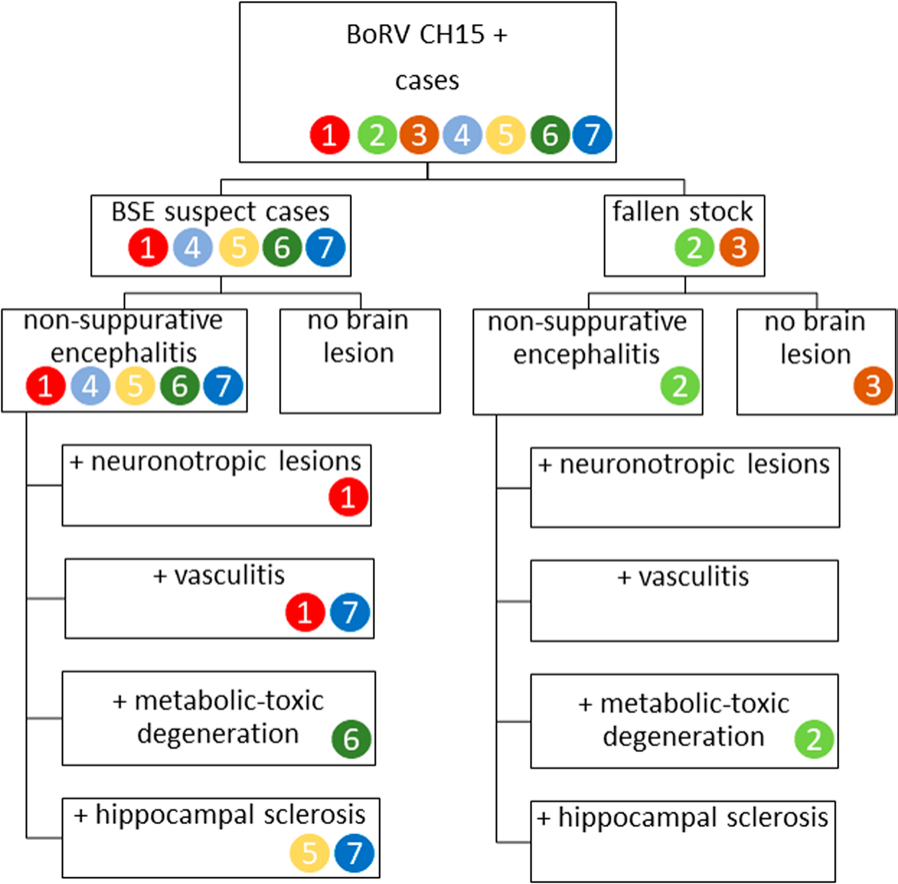
Overview of lesion types observed this study. Case numbers are in colored circles. BoRV CH15, bovine retrovirus CH15; BSE, bovine spongiform encephalopathy

All cases showed clear non-suppurative encephalitis, with the exception of case 3, which had no histological abnormality in the caudal brainstem, the only tissue available. Non-suppurative encephalitis was characterized by gliosis and mononuclear cell infiltrates, the latter presenting mainly as perivascular cuffs (Fig. 3A, C, D). These lesions had a multifocal distribution. In one animal (case 1), neuronotropic lesions comprised of glial nodules around neurons, a typical manifestation of neuronal viral infections, were present (Fig. 3B). In two animals (cases 1 and 7), mononuclear cells were not only located perivascularly in the Virchow-Robin space, but also infiltrated the vessel wall, i.e., these animals exhibited lymphohistiocytic vasculitis (Fig. 3C). Additionally, severe and acute ecchymotic and ring hemorrhages were seen multifocally in the brain of one of these animals (case 7; Fig. 3D). The cause of these lesions could not be determined clearly; they could have occurred secondary to vascular changes, but also partly due to captive-bolt stunning of the animal. In two animals (cases 2 and 6), severe diffuse neuronal degeneration was present, manifesting with chromatolytic, swollen, and eosinophilic neurons (Fig. 3E). In case 6, for which multiple brain regions were available for histopathological examination, degenerated neurons were found in the motoneurons of cranial nerve, basal, red, and thalamic nuclei, as well as in the Purkinje cells. Additionally, peri- and intraneuronal vacuolization was observed in the cerebral cortex. Neuronal changes were multifocal, but not always associated with perivascular cuffs, and gliotic nodules surrounding the altered neurons were absent. Taken together, these findings suggest a metabolic-toxic event as the cause of the widespread neuronal degeneration. In two other animals (cases 5 and 7), hippocampal sclerosis with chromatolysis and the degeneration of pyramidal cells surrounded by hypertrophic astrocytes was observed (Fig. 3F). These lesions were not in association with perivascular infiltrates. An overview of lesion types in the animals examined in this study is provided in Fig. 4.

**Fig. 5:**
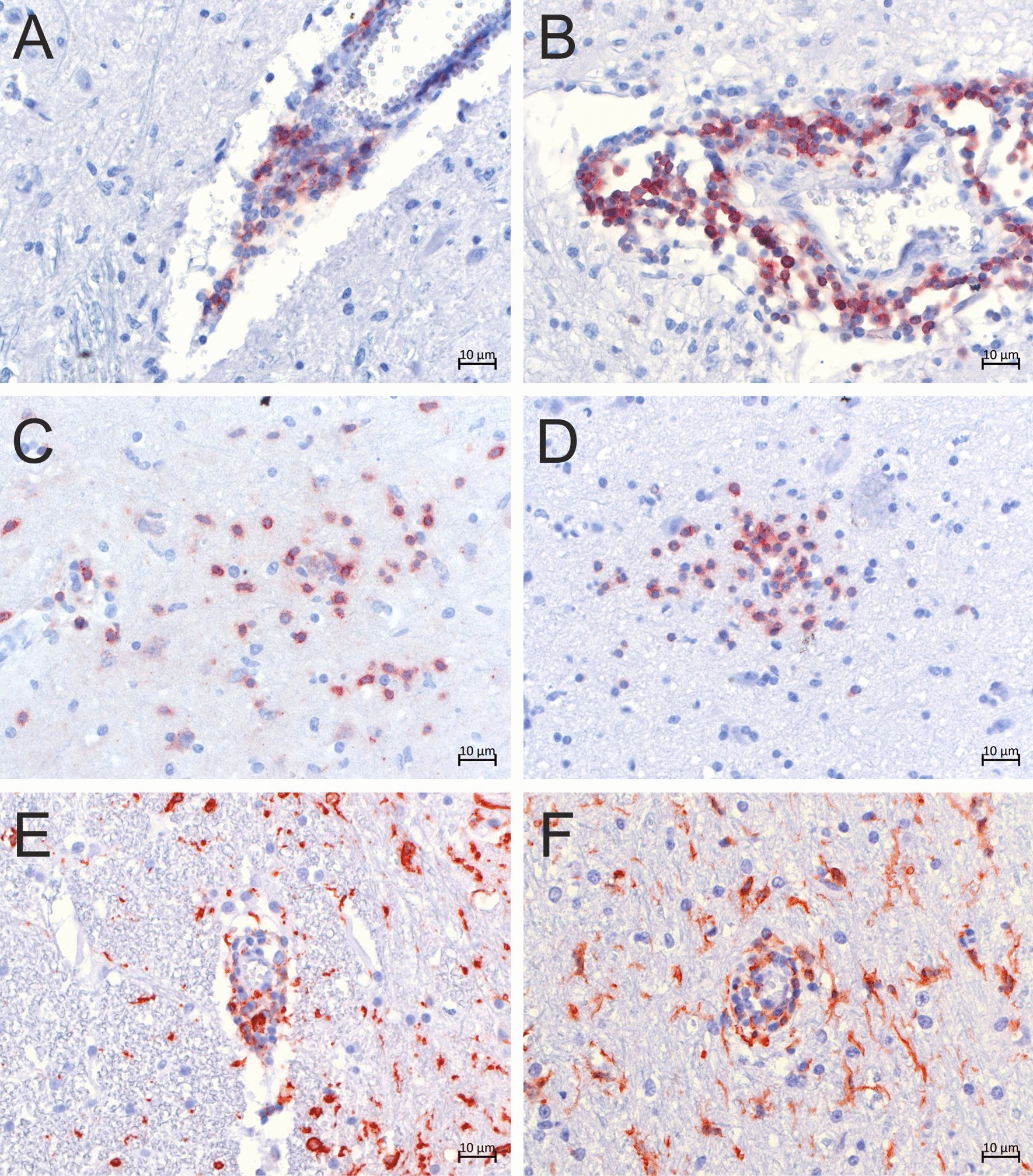
T-lymphocytes and macrophages contributed in high numbers to perivascular cuffs and gliosis. **A**–**D** Immunohistochemical analysis was performed with staining with an antibody targeting CD3. Positivity (red granular staining) is visible in the cytoplasm of CD3 + T-lymphocytes in perivascular cuffs (**A** case 6, medulla oblongata; **B** case 5, thalamus), diffuse gliosis (**C** case 6, medulla oblongata), and nodular gliosis (**D** case 6, hypothalamus). **E**, **F** Immunohistochemical analysis was performed with staining with an antibody targeting ionized calcium-binding adapter molecule 1. Positivity (red granular staining) is visible in the cytoplasm of cells in perivascular cuffs and ramified cells in the neuroparenchyma (microglia; **E** case 2, medulla oblongata; **F** case 4, thalamus)

We performed immunohistochemistry (IHC) with antibodies labeling CD3+ T-lymphocytes to demonstrate the involvement of the adaptive immune system in non-suppurative encephalitis in the cases under investigation. High numbers of CD3+ T-lymphocytes contributed to perivascular cuffs in four animals (cases 1, 5, 6, and 7; Fig. 5A and B) and were additionally found in the surrounding parenchyma in diffuse and nodular gliosis (Fig. 5C and D). The involvement of the adaptive immune system in case 6, in which lesion type and distribution suggest the occurrence of a metabolic-toxic event and not the involvement of pathogens (Fig. 5A), was unexpected. In the perivascular cuffs of the other two animals (cases 2 and 4), however, the predominant cell type was not T-lymphocytes, but ionized calcium-binding adapter molecule 1 (Iba-1)-positive macrophages (Fig. 5E and F).

### In situ detection of bovine retrovirus CH15

**Fig. 6:**
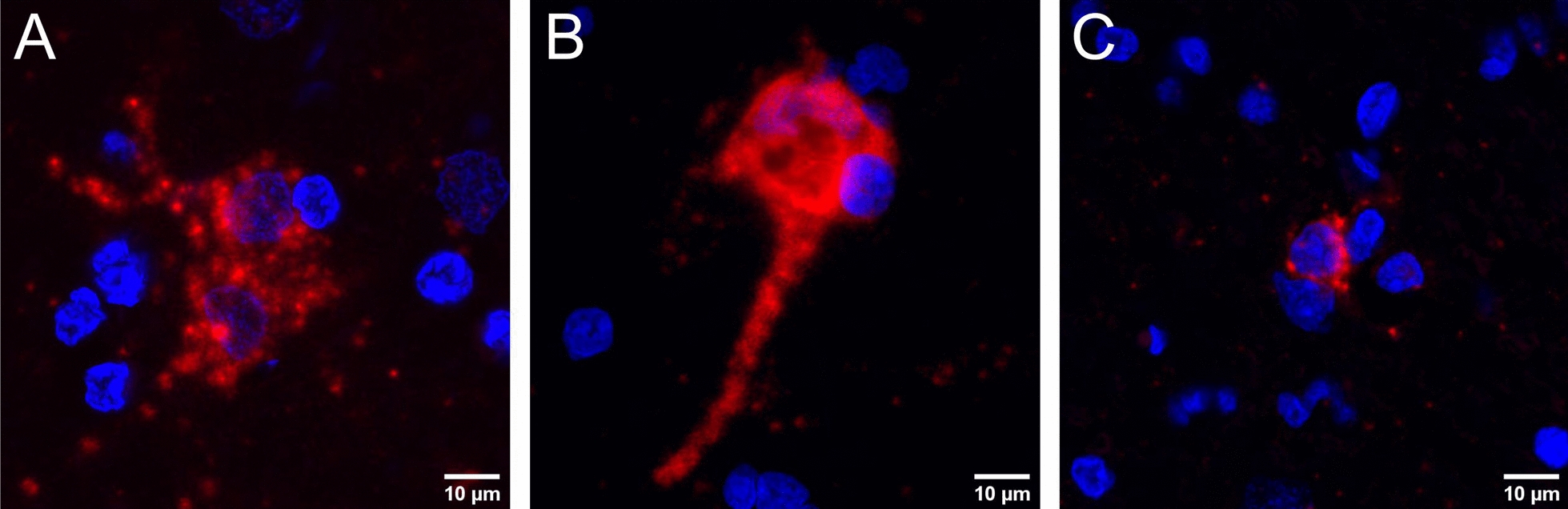
Detection of bovine retrovirus CH15 (BoRV CH15) RNA in situ in the cellular cytoplasm. Fluorescent in situ hybridization of formalin-fixed, paraffin-embedded tissue slides using the BoRV CH15 RNAscope probe. Nuclei are stained in blue, BoRV CH15 RNA is stained in red. **A** Case 1, medulla oblongata; **B** case 6, cerebral cortex; **C** case 7, medulla oblongata. The morphology of the positive cell in **B** is consistent with a neuron

**Fig. 7:**
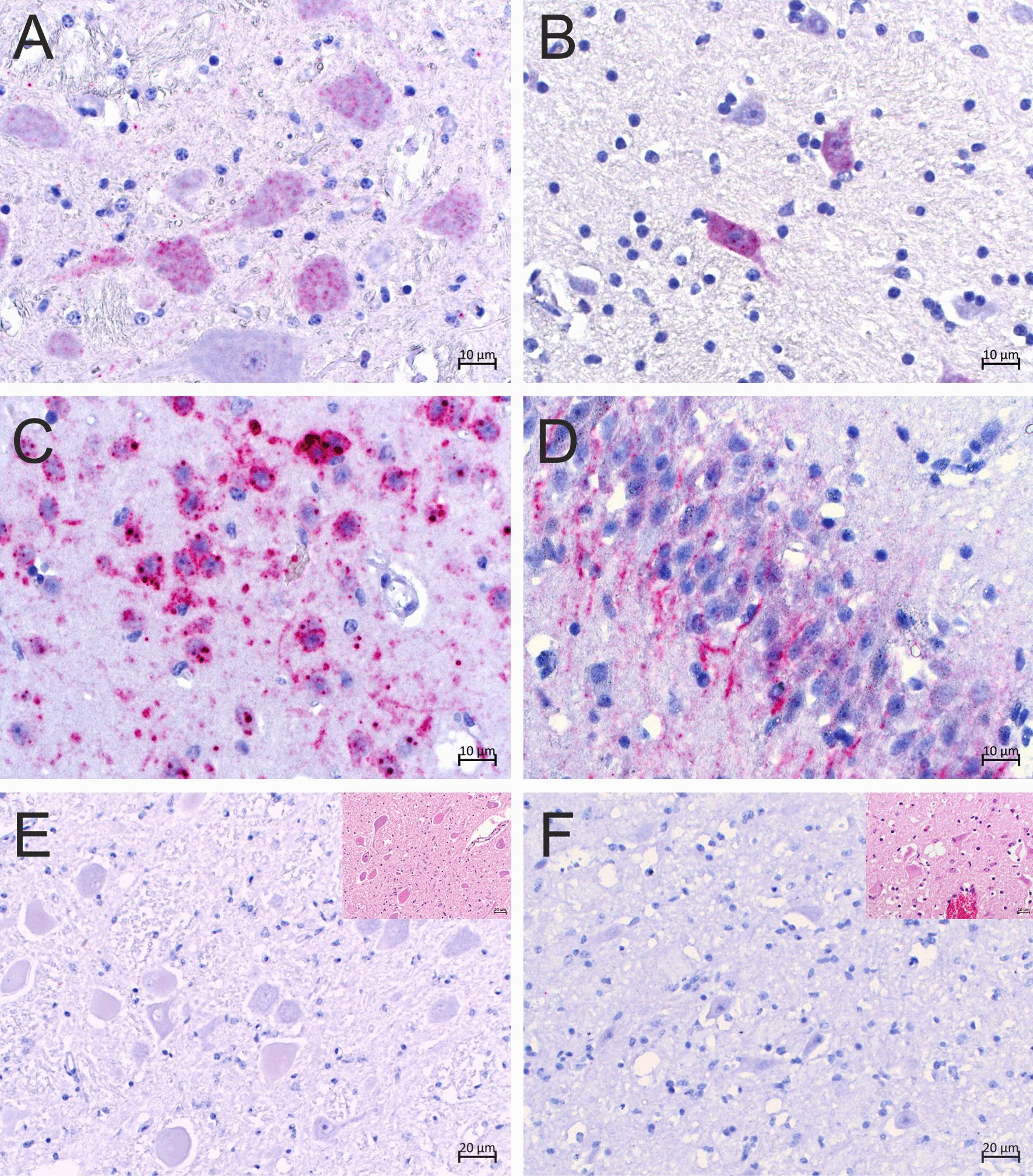
Detection of bovine retrovirus CH15 (BoRV CH15) RNA in cells morphologically compatible with neurons. Chromogenic in situ hybridization of formalin-fixed, paraffin-embedded tissue slides using the BoRV CH15 RNAscope probe. BoRV CH15 RNA (red granular staining) is visible in the cytoplasm of cells and cellular extensions morphologically compatible with neurons. The neurons appear to be morphologically normal, with the maintenance of Nissl substance, and inflammatory cells are absent from the immediate surroundings of positive neurons. **A** Case 1, medulla oblongata; **B** case 6, hypothalamus; **C** case 6, cerebral cortex; **D** case 7, hippocampus. Neurons with clear signs of necrosis (chromatolytic and eosinophilic cytoplasm, eccentric nuclei, loss of Nissl substance) on hematoxylin and eosin staining (insets) remained negative for BoRV CH15 RNA. **E** Case 6, medulla oblongata; **F** case 7, hippocampus

For in situ detection of BoRV CH15 RNA, fluorescent and chromogenic in situ hybridization (ISH) was performed on formalin-fixed, paraffin-embedded (FFPE) brain tissue slides. The probe used for ISH targeted the gag ORF; more details can be found in the materials and methods section. For cases 2 and 3, only medulla oblongata material was available. For the remaining cases, brain regions showing typical signs of non-suppurative encephalitis on hematoxylin and eosin (H&E) staining were used. Fluorescent ISH enabled the detection of viral RNA with a clear cytoplasmic distribution in five animals (cases 1, 2, 4, 6, and 7; Fig. 6A–C). In one of these animals (case 6), the morphology of the infected cells was consistent with neurons (Fig. 6B). To assess the infected cell type in the remaining positive animals, chromogenic ISH and counterstaining with Mayer's hemalum solution were performed. This analysis enabled the detection of BoRV CH15 RNA, mostly in cells morphologically compatible with neurons (Fig. 7A–C) and in cellular extensions of neurons (Fig. 7D). Labeling was located in brain areas with lesions of non-suppurative encephalitis, but with no clear topographic association of nodular or diffuse gliosis with BoRV CH15 RNA. BoRV CH15 RNA labeling was absent in highly eosinophilic and chromatolytic neurons in cases 2 and 6 (Fig. 7E), in which metabolic-toxic events were believed to have caused the pathological changes, and in pyramidal cells of the hippocampus in cases 5 and 7 (Fig. 7F), although neuronal extensions of presumably granular cells in the hippocampus were strongly positive in case 7 (Fig. 7D). In two animals (cases 3 and 5), we could not identify BoRV CH15 RNA in situ in the available material. Negative control FFPE tissue slides from animals with non-suppurative encephalitis and BoRV CH15 negativity remained negative in both ISH staining analyses.

## Discussion

In this study, we describe new full-length or nearly-full-length viral genomes that are highly similar to the recently described BoRV CH15 obtained from the brain tissue of neurologically diseased cows. All viral genomes are closely related and phylogenetically affiliated to the genus *Betaretrovirus*. Still, they show up to 11.5% variation among each other at the coding-sequence level. Nucleotide differences are partly synonymus, as seen in the higher percentage of identity on protein level indicating conservative pressure in the coding regions. However, this variance is the first argument that the BoRV CH15 sequence might belong to an exogenous retrovirus and does not represent endogenous viral elements. Phylogenetic analysis also showed a distant relation of BoRV CH15 to known bovine endogenous retroviruses on the nucleotide level. Additionally, BoRV CH15 was found in animals of different breeds but except for Tyrol Grey (a breed not represented among the control animals) not in healthy slaughtered control animals of the same breeds, which is another argument against its characterization as an endogenous retrovirus. The differentiation of exogenous and endogenous retroviruses is crucial for estimation of the relevance of BoRV CH15, especially with regard to acute disease association. Endogenous retroviral sequences are incorporated in the host chromosomal genome at a specific evolutionary timepoint, and integration into the germline leads to the maintenance of the genetic information in the species [29]; thus, they do not reflect an acute infection event and are mostly unrelated to disease. However, endogenous retrovirus proteins can also be expressed, and in humans endogenous retroviruses have been discussed to be associated with multiple sclerosis and amyotrophic lateral sclerosis [30–32]. Another argument for active viral infection was obtained by sequencing RNA extracted from brain material; we were able to determine coding-complete viral genome sequences for all BoRV CH15–positive animals exhibiting non-suppurative encephalitis and neurological signs in this study (cases 1, 2, 4, 5, 6, and 7). The detection of abundant viral RNA and/or mRNA in these animals indicates active infection with viral transcription and replication, although restricted viral replication without the production of infectious particles leading to pyroptosis in infected CD4 T-cells has been described for HIV [33, 34]. Taken together, these arguments support the classification of the BoRV CH15 strains as exogenous infectious retroviruses. However, this does not definitely disprove BoRV CH15 as an activated and expressed endogenous retrovirus.

In the one animal with no pathological brain lesion (case 3), we could not obtain the coding-complete sequence of BoRV CH15 through HTS RNA sequencing. However, we determined nearly the entire coding-complete viral sequence from extracted DNA. The different outcomes of RNA and DNA sequencing reflect low levels of viral transcription and replication. The absence of pathological lesions suggests that the brain infection had occurred shortly before the animal’s death and did not progress to the extent of pathological change, or that the animal stayed asymptomatic even though the infection took place already some time ago, as it known for other retroviruses [35–37]. However, latent infection of blood cells also could have led to the detection of BoRV CH15 in brain tissue extracts, mimicking brain infection, as brain material always contains a certain amount of blood. The inability to detect BoRV CH15 RNA in situ in the brain of this animal further supports this hypothesis. However, only the brainstem was available for nucleic acid extraction, pathological examination, and in situ detection; thus, active infection of other brain regions cannot be excluded.

Our attempt to elucidate the association of BoRV CH15 with neurological clinical signs was hampered by a lack of comprehensive clinical information. In six of the seven BoRV CH15–positive animals, neurological signs were reported, but without uniform manifestation. Similarly, the observed histopathological lesions varied among the animals. Although all animals with neurological signs were diagnosed with non-suppurative encephalitis and BoRV CH15 showed clear neuronotropism, neuronotropic lesions were observed in only one animal (case 1). Four of the six animals with non-suppurative encephalitis had severe brain lesions that were interpreted to be additional to the inflammatory lesions (neuronal degeneration of the metabolic-toxic type and hippocampal sclerosis). Thus, these lesions were likely not related directly to BoRV CH15 infection. Additionally, the virus was not found in the degenerated and sclerosing neurons, supporting the lack of the lesions’ relationship to infection. Neuronal degeneration due to metabolic-toxic events can lead to unspecific, but severe, neurological signs in cattle, and metabolic-toxic insults must be considered as alternative or additional causes for the clinical manifestations in these animals. Hippocampal sclerosis is a known phenomenon in humans, cats, and likely dogs; it manifests as temporal lobe epilepsy (TLE), but can have a variety of etiologies [38–40]. In humans, viral encephalitis has been discussed as a possible TLE-predisposing factor along with febrile seizures, genetic predisposition, hippocampal malformation, brain injury, autoimmune responses, and many other factors [38, 41]. Affected individuals show seizures and, indeed, neurological signs reported for one animal with comparable lesions in this study (case 5) were described as seizure-like. The combination of non-suppurative encephalitis and hippocampal sclerosis has been described in cattle with neurological disease in the UK and designated “idiopathic brainstem neuronal chromatolysis and hippocampal sclerosis (IBNC)” of cattle, for which the etiology remains unresolved [42]. The possible involvement of viral infection in the hippocampal sclerosis observed in the cows in this study cannot be excluded, but is certainly not the only probable explanation, especially in comparison with TLE. It would be interesting to test cases of IBNC for BoRV CH15 infection.

On unbiased HTS, BoRV CH15 was the only virus discovered in all positive animals, which argues for a causative relationship between BoRV CH15 and non-suppurative encephalitis. However, the HTS sequencing may have missed novel, highly divergent viruses. In contrast to metabolic-toxic degeneration and hippocampal sclerosis, a viral etiology is likely for neuronotropic lesions and vasculitis, as seen in cases 1 and 7. However, although ISH revealed strong neurotropism for BoRV CH15, we could not demonstrate viral RNA in any kind of lesion—neither in close association with activated inflammatory cells in lesions of non-suppurative encephalitis nor in neurons showing degeneration due to metabolic-toxic changes or hippocampal sclerosis—which brings the causative association of BoRV CH15 with neuropathology into question. On the other hand, BoRV CH15 was detected exclusively in diseased cattle and not in healthy slaughtered animals, and viral RNA was demonstrated in five  of the six cases with brain lesions in situ, even if not in direct association with inflammatory cells or altered neurons, in the same brain areas where signs of non-suppurative encephalitis were detected. In case 5, detection of BoRV CH15 was not possible in any brain region with histopathological lesions, but the variance between the gag ORF used for ISH probe design and the corresponding gag ORF sequenced from this animal was high (11.6%) and could be the reason for the negative ISH result.

The presence of the NF-1 transcription factor–binding site in the LTR of the BoRV CH15 retroviruses is compatible with the transcriptional activity observed in neuronal cells. The transcription factor NF-1 is active in neurons and plays a crucial role in driving the expression of neuron-specific gene products, such as neurofilament proteins. These neurofilaments play an essential role in determining the volume and form of neurons [43]. NF-1 transcription factor–binding sites are also present in the LTRs of several retroviruses, such as the Moloney murine leukemia virus [44]. The presence of this site in the BoRV CH15 genomes is, thus, in line with the observed neuronotropism of BoRV CH15.

Overall, the association of BoRV CH15 infection with non-suppurative encephalitis and neurological disease in cattle cannot be confirmed or ruled out at this time. More research is needed to estimate the importance of this virus for the bovine population. Such research may include experimental infection studies once the virus has been isolated in cell culture. The first step would be the detection of viral particles by electron microscopy. Unfortunately, formalin fixation is suboptimal for electron microscopy and, therefore, we did not apply this technique on our FFPE tissue. Our attempts to propagate the virus in different cell lines of bovine origin have been unsuccessful (data not shown), likely due to reduced virus infectivity because of advanced tissue autolysis prior to sampling and suboptimal prolonged storage conditions. Establishment of primary cultures of apparent CNS target cells would be another important step towards the propagation of the virus in cell culture. Due to poor tissue conditions of the brain material of this study it was not performed until now. Nonetheless, these are important directions to estimate the association of BoRV CH15 to disease in cattle and should be included in further investigations.

Notably, not all retroviruses, and especially not all bovine retroviruses, are strictly pathogenic. Upon infection with BIV, for example, mild lymphocytosis, generalized hyperplasia of the lymph nodes, and mild lymphocytic perivascular cuffs have been described, but without clinical manifestation in taurine cattle [45–47]. A similar scenario is conceivable for BoRV CH15, with infection leading only to non-suppurative encephalitis and the neurological clinical signs and characteristic neuropathological lesions described in our cases having a different cause. The high mean age (10.8 years) of the positive animals in this study favors a long incubation period before brain manifestations appear, which is in line with knowledge of retrovirus infections [48, 49] and increases the probability that the animals had additional illnesses. BoRV CH15 showed a close phylogenetic relationship to tumor-causing betaretroviruses, but not to members of the genera *Lentivirus*, *Gammaretrovirus*, or *Deltaretrovirus*, which are retroviruses with neuroinvasive potential. The infected cell type, observed pathological lesions, and/or topographic location of CNS infection differ between BoRV CH15 infection and infection with known neuropathogenic retroviruses. Lentivirus infection can result in a similar brain lesion pattern, with gliosis, perivascular infiltration, and neuronal loss, but the target cells are microglia, whereas BoRV CH15 infects neurons. Additionally, demyelination with subsequent malacia occurs after lentivirus infection, which was not seen in the brains of the animals of this study [25, 50–53]. Gammaretroviruses infect different cell types, including glia and endothelial cells, but also neurons, as does BoRV CH15. However, infection with these viruses leads mainly to spongiform degeneration, which was not present in BoRV CH15–infected brains [25, 54]. Deltaretrovirus infection is typically restricted to the spinal cord, and the infected cells are mainly lymphocytes [25, 55]. Unfortunately, the spinal cords of the animals examined in this study were not available for investigation. Further screening studies with blood and organs other than the brain, and serological surveys based, for example, on a recombinant Gag protein, must be performed to gain more information about the distribution of BoRV CH15 in the cattle population, the association of this virus with disease, and possible body reservoirs. The incidence of BoRV CH15 infection, especially in diseased cows, should be monitored to detect a sudden change in pathogenicity accompanied by a rise in infection frequency at an early stage.

For investigations of the host range, viral life cycle, and possible pathomechanisms of BoRV CH15, a suitable in vitro system is required. DNA sequencing enabled determination of the complete LTRs of six BoRV CH15 genomes and description of their full-length provirus sequences, which can serve as the basis for the construction of a molecular clone. Molecular clones are powerful tools for retrovirus research [56, 57] and, thus, have the potential to form the basis of in vitro investigations of BoRV CH15.

## Conclusions

In this study, we described seven full-length or nearly full-length BoRV CH15 genomes and investigated their associations with neurological disease in cattle. Although we could not indisputably confirm or exclude a causative relationship of BoRV CH15 to disease, we raise awareness of this new retrovirus in cattle and our findings provide the basis for further in vitro investigations.

## Materials and methods

### Samples

For this study, FFPE brain tissue slides (native and stained with H&E) and fresh-frozen brain material including at least the cerebral cortex, medulla oblongata, thalamus, midbrain, cerebrum, and hippocampus were available from cases 1, 4, 5, 6, and 7. From cases 2 and 3, only FFPE brain tissue slides (native and stained with H&E) and fresh-frozen medulla oblongata material was available. The slides had been neuropathologically analyzed at the Division of Neurological Sciences, University of Bern, Switzerland, upon case submission. Fresh-frozen medulla oblongata material collected from healthy slaughtered control animals originating from two slaughterhouses in Zurich and Estavayer, Switzerland, for use in a previous study [9] was also examined in this study. One hundred and thirty brain samples, in which the absence of histopathological lesions was confirmed at the Division of Neurological Sciences, University of Bern, Switzerland, as part of the previous study, were selected for further analysis. FFPE material and DNA extracted from negative control animals with non-suppurative encephalitis of unknown origin, but with negative HTS results for BoRV CH15 [8], was also available for analysis.

### Nucleic acid extraction

RNA and DNA were extracted from the brain tissues of BoRV CH15–positive animals with pooling of areas of the frontal cortex, thalamus, and medulla oblongata of individual animals. RNA was extracted with TRI Reagent (Sigma Life Sciences, St. Louis, MO, USA) and DNA was extracted with the DNeasy Blood and Tissue Kit (Qiagen, Hilden, Germany) according to the manufacturers' instructions. For DNA extraction from brain tissues of healthy slaughtered control animals, fresh-frozen medulla oblongata was used. Extraction was performed on the Maxwell® RSC 48 instrument (Promega Corporation, Madison, WI, USA) using the Maxwell® RSC PureFood GMO and Authentication Kit for Food, Feed and Seed Samples (Promega Corporation) according to the manufacturer's instructions.

### High-throughput sequencing

For HTS, total RNA extracts of pooled medulla oblongata, thalamus, and cerebral cortex (cases 4–7) or medulla oblongata alone (case 3) were used to prepare sequencing libraries with the TruSeq Stranded Total RNA kit (Illumina Inc., San Diego, CA, USA) according to the manufacturer's instructions. The libraries were then sequenced on an Illumina HiSeq 3000 or Illumina NovaSeq 6000 machine (Illumina Inc.) in paired-end mode with 2 × 100 or 2 × 150 cycles (Additional file 1).

### Bioinformatics analysis

In general, the sequence analysis pipeline was as described previously [58]. Briefly, raw reads were quality selected with FastQC (ver. 0.11.7; [59]), trimmed with fastp (ver. 0.12.5; [60]), and mapped to the *Bos taurus* reference genome (BioProject PRJNA32899 Bos_taurus.UMD3.1.dna.toplevel) using STAR (ver. 2.5.3a; [61]). Quality-selected unmapped reads were assembled using SPAdes (ver. 3.10.1; [62]) and the resulting scaffolds were compared to virus databases (GenBank viral nucleotide sequences, 22 May 2020; UniProt viral amino-acid sequences, 10 May 2020) using BLASTN (ver. 2.6.0 + ; [63]) and DIAMOND (ver. 0.9.19; [64]). The U3 sequences were analyzed using AliBaba2.1 software [65] with the following setup: pairsim to known sites = 64, mat. width = 10 bp, and min mat. conservation = 80% (high).

### Bovine retrovirus CH15 PCR

PCR was performed with 1× GoTaq Green Mastermix (Promega Corporation) with previously published primer pairs and corresponding protocols [8]. The primers amplified 500 bp of the gag ORF (BoRV_GAG_L/BoRV_GAG_R), 318 bp of the pol ORF (BoRV_POL_L/BoRV_POL_R), and 382 bp of the env ORF (BoRV_ENV_L/BoRV_ENV_R). The PCR products were analyzed on 1% agarose gels. Water samples and DNA extracted from animals with non-suppurative encephalitis but confirmed BoRV CH15 negativity served as negative controls. DNA extracted from index animal 25018 (case 1) served as a positive control [8].

### Bovine 12s rDNA qPCR

Quantitative PCR targeting the bovine 12s rDNA was performed with Power SYBR™ Green PCR Master Mix (Thermo Fisher Scientific Inc., Waltham, MA, USA) and primers published elsewhere [66]. The reaction contained 1× Power SYBR™ Green PCR Master Mix, 600 nM of each primer, 2 μl DNA and nuclease-free water up to 25 μl. The qPCR was run on a CFX96TM Real Time System (BioRad, Hercules, CA, USA). Cycling conditions included an initial phase of 10 min at 95 °C, followed by 40 cycles of 15 s at 95 °C and 1 min at 60 °C. After the amplification step, a melting curve was performed starting at 60 °C, incrementally increasing by 0.5 °C until 95 °C was reached. Data were analyzed using the CFX Maestro software (version 4.1.2433.1219; BioRad).

### Sanger sequencing

For Sanger sequencing of viral RNA, a reverse transcription step using SuperScript™ III reverse transcriptase (Thermo Fisher Scientific Inc.) was first performed with random hexamers. The resulting cDNA or extracted DNA for sequencing of the proviral DNA was amplified with primers designed with the Geneious Prime software (ver. 2020.2.4; Biomatter, Auckland, New Zealand) and Q5® Hot Start high-fidelity DNA polymerase (New England Biolabs Inc., Ipswich, MA, USA). All primers used for sequencing are listed in Additional file 6. After gel electrophoresis and purification with NucleoSpin® gel and a PCR Clean-up Kit (Macherey–Nagel, Düren, Germany), 3 µl purified PCR product was used for Sanger sequencing in a 3730 DNA analyzer (Thermo Fisher Scientific Inc.) with the BigDye® Terminator v3.1 Cycle Sequencing Kit (Thermo Fisher Scientific Inc.). Standard protocols were used for all experiments. The sequencing data generated were analyzed using the Geneious Prime software (ver. 2020.4.2; Biomatter).

### Rapid amplification of cDNA ends

For RACE, the SMARTer® RACE 5'/3' Kit (Takara Bio Inc., Kusatsu, Japan) was used according to standard protocols and with gene-specific primers binding 105 and 28 bp upstream of the env ORF 3' end. Five-prime RACE was performed with the SMARTer® RACE 5'/3' Kit (Takara Bio Inc.) and the 5' RACE System Kit (Thermo Fisher Scientific Inc.), with gene-specific primers binding 82 and 23 bp downstream of the gag ORF 5' end. The RACE products were separated by gel electrophoresis, purified, and Sanger sequenced as described above.

### Phylogenetic comparisons

For phylogenetic analysis, coding sequences of representative members of the exogenous viruses of the genera *Betaretrovirus*, *Lentivirus*, *Gammaretrovirus*, *Deltaretrovirus,* and *Bovisumavirus,* as well as the env mRNA of bovine endogenous retroviruses were aligned with mafft using the FFTNS-NS-2 method (ver. 7.475, [67]). A phylogenetic tree was constructed using iqtree2 (ver. 2.0.3, [68]) with the GTR + F + R3 model and 1′000 bootstraps, and edited using MEGA-X [69].

### Immunohistochemistry

For all animals with typical lesions of non-suppurative encephalitis, FFPE brain tissue slides were labeled with antibodies targeting CD3 and Iba-1 by IHC. CD3 labeling was performed as described elsewhere [70]. Briefly, the slides were deparaffinized and rehydrated, and endogenous peroxidase activity was blocked with 3% H_2_O_2_ in methanol. Antigen retrieval was performed with the Target Retrieval Solution (pH 6) of the RNAscope® System (Advanced Cell Diagnostics, Newark, NJ, USA) as described by the manufacturer. The polyclonal rabbit anti-human CD3 antibody (Dako Denmark A/S, Glostrup, Denmark) was diluted 1:200 in Dako antibody diluent (Dako Denmark A/S) and incubated at 4 °C overnight. Subsequently, the signal was detected with the Mouse and Rabbit Specific HRP/DAB (ABC) Detection IHC kit (ab64264; Abcam plc, Cambridge, UK) with standard protocols, and the slides were counterstained with Mayer's hemalum solution (Merck KGaA, Darmstadt, Germany) and mounted with Aquatex® medium (Merck KGaA). The working procedures for immunohistochemical labeling of Iba-1 followed the same standard protocol as those for CD3 labeling, with minor modifications. Antigen retrieval was performed in Dako REAL Retrival Solution (pH 9; Dako Denmark A/S) for 20 min at 95 °C in an H2850 Microwave Processor (EBSciences, East Granby, CT, USA). The polyclonal rabbit anti–Iba-1 antibody (WAKO, Chuo-ku, Osaka, Japan) was diluted 1:500 in phosphate-buffered saline containing 0.5% Tween (PBS-T) and incubated for 1 h at 37 °C. All slides were analyzed under a Zeiss Axio Scope.A1 microscope (Carl Zeiss Microscopy GmbH, Göttingen, Germany).

### In situ hybridization

An ISH probe (V-BoRV-CH15-27214987) targeting the positive strand of the gag ORF of BoRV CH15 (nt position 561–1′551 of NC_029852.1; cat no. 1031811-C1) was ordered from Advanced Cell Diagnostics. The FFPE brain tissue slides used for IHC were also used for ISH. Fluorescent and chromogenic ISH was performed with the RNAscope® 2.5 HD Reagent Kit-RED (Advanced Cell Diagnostics) according to the manufacturer's instructions. Fluorescent ISH slides were counterstained with Dapi BioChemica (AppliChem GmbH, Darmstadt, Germany) diluted 1:10′000 in PBS-T for 1 h at room temperature in humid conditions. The slides were mounted with Glycergel® aqueous mounting medium (Dako Denmark A/S) and analyzed under an Olympus Fluoview FV3000 Confocal Laser 361 Scanning Microscope (Olympus Europa, Hamburg, Germany). Chromogenic ISH slides were counterstained with Mayer's hemalum solution (Merck KGaA), mounted with Aquatex® medium (Merck KGaA), and analyzed under a Zeiss Axio Scope.A1 microscope (Carl Zeiss Microscopy GmbH).

## Supplementary Information

**Additional file 1:** Details of high-throughput sequencing runs and BoRV CH15–specific scaffolds in cases 1 to 7.

**Additional file 2:** Read pairs with one mate mapping to the *Bos taurus* genome and one mapping to BoRV CH15.

**Additional file 3:** Possible insertions in the long terminal repeats of BoRV CH15 genomes of case 5 and 7.

**Additional file 4:** Alignment of the six BoRV CH15 unique 3' sequences. Alignment with Geneious Prime software (ver. 2020.4.2) revealed a conserved nuclear factor 1 binding site that potentially controls the peculiar, neuron-specific transcriptional activity of these retroviruses.

**Additional file 5:** Pairwise identities (%) of BoRV CH15 genome elements.

**Additional file 6:** Primers used for Sanger sequencing.

## Data Availability

Sequencing raw reads are available from the NCBI short read archive: Bioproject PRJNA743804. Proviral genome sequences are available from GenBank (accession numbers KU720628 [case 1], OM339153 [case 2], OM339154 [case 3], OM339155 [case 4], OM339156 [case 6], OM339157 [case 7] and OM339158 [case 5].
