# Correction to: A novel Betaretrovirus discovered in cattle with neurological disease and encephalitis

**DOI:** 10.1186/s12977-022-00590-8

**Published:** 2022-02-07

**Authors:** Melanie M. Hierweger, Michel C. Koch, Ronja V. Kauer, Zoltán Bagó, Anna Oevermann, Giuseppe Bertoni, Torsten Seuberlich

**Affiliations:** 1 Division of Neurological Sciences, Department of Clinical Research and Veterinary Public Health, Vetsuisse Faculty, University of Bern, Bern, Switzerland; 2 Austrian Agency for Health and Food Safety (AGES) Ltd., Institute for Veterinary Disease Control, Mödling, Austria; 3 Institute of Virology and Immunology, Bern, Switzerland; 4 Department of Infectious Diseases and Pathobiology, Vetsuisse Faculty, University of Bern, Bern, Switzerland

## Correction to: Retrovirology (2021) 18:40 https://doi.org/10.1186/s12977-021-00585-x

Following publication of the original article [1], it was reported that the text did not contain reference to the NCBI GenBank entries of the proviral genome sequences determined in this study.

The ‘Availability of data and materials’ declaration should include the statement "Proviral genome sequences are available from GenBank (accession numbers KU720628 [case 1], OM339153 [case 2], OM339154 [case 3], OM339155 [case 4], OM339156 [case 6], OM339157 [case 7] and OM339158 [case 5]."

The original article [1] has been corrected.
