# Supplementary figures and images for: A novel Betaretrovirus discovered in cattle with neurological disease and encephalitis
Source: Retrovirology. 2021 Dec 20;18:40. doi: 10.1186/s12977-021-00585-x (PMC8686636; doi:10.1186/s12977-021-00585-x)

# Additional file 4: Alignment of the six BoRV CH15 unique 3' sequences

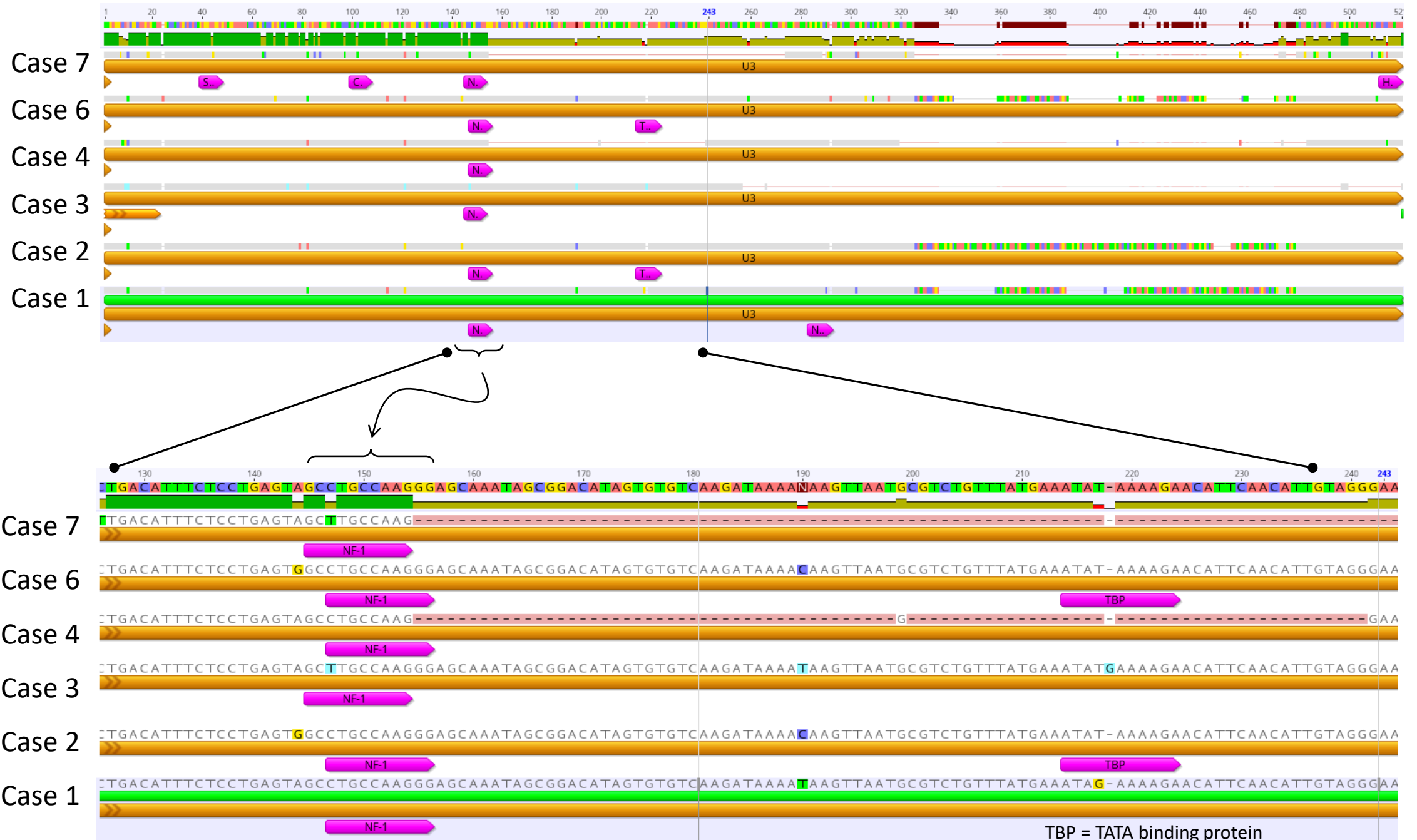

Supplement: Supplementary file 4 — Additional file 4: Alignment of the six BoRV CH15 unique 3' sequences. Alignment with Geneious Prime software (ver. 2020.4.2) revealed a conserved nuclear factor 1 binding site that potentially controls the peculiar, neuron-specific transcriptional activity of these retroviruses. [file 12977_2021_585_MOESM4_ESM.pdf]
